# Can Schools Reduce Adolescent Psychological Stress? A Multilevel Meta-Analysis of the Effectiveness of School-Based Intervention Programs

**DOI:** 10.1007/s10964-020-01201-5

**Published:** 2020-02-07

**Authors:** Amanda W. G. van Loon, Hanneke E. Creemers, Wieke Y. Beumer, Ana Okorn, Simone Vogelaar, Nadira Saab, Anne C. Miers, P. Michiel Westenberg, Jessica J. Asscher

**Affiliations:** 1 Utrecht University Child and Adolescent Studies, Heidelberglaan 1, 3584 CS Utrecht, The Netherlands; 2 University of Amsterdam Forensic Child and Youth Care Sciences, Nieuwe Achtergracht 127, 1018 WS Amsterdam, The Netherlands; 3 Leiden University Developmental and Educational Psychology, Wassenaarseweg 52, 2333 AK Leiden, The Netherlands; 4 Leiden University Graduate School of Teaching (ICLON), Kolffpad 1, 2333 BN Leiden, The Netherlands

**Keywords:** Psychological stress, Meta-analysis, Adolescent, School-based intervention programs

## Abstract

Increased levels of psychological stress during adolescence have been associated with a decline in academic performance, school dropout and increased risk of mental health problems. Intervening during this developmental period may prevent these problems. The school environment seems particularly suitable for interventions and over the past decade, various school-based stress reduction programs have been developed. The present study aims to evaluate the results of (quasi-)experimental studies on the effectiveness of school-based intervention programs targeting adolescent psychological stress and to investigate moderators of effectiveness. A three-level random effects meta-analytic model was conducted. The search resulted in the inclusion of *k* = 54 studies, reporting on analyses in 61 independent samples, yielding 123 effect sizes (*N* = 16,475 individuals). The results indicated a moderate overall effect on psychological stress. Yet, significant effects were only found in selected student samples. School-based intervention programs targeting selected adolescents have the potential to reduce psychological stress. Recommendations for practice, policy and future research are discussed.

## Introduction

Stress—the condition or feeling that results when individuals perceive that the demands of a situation exceed their personal, psychological or social resources (Lazarus 1966)—seems to be a significant worldwide problem for both adolescents (Klinger et al. 2015) and adults (Schaufeli et al. 2009). In adolescence, a developmental period characterized by increased stress-sensitivity (Romeo 2013), high levels of stress have been linked to various negative associates, including reductions in academic performance (Kaplan et al. 2005), school drop-out (Dupéré et al. 2015), increased mental health problems (Snyder et al. 2017), and reduced well-being (Chappel et al. 2014). In order to prevent adverse development, it is important to address heightened stress levels during adolescence. Over the past decade, various school-based intervention programs have been implemented to reduce adolescent stress and accompanying effectiveness studies have been conducted. As knowledge on the overall effectiveness of such programs and factors influencing their effectiveness is limited, it is important to conduct an extensive meta-analysis. The current multilevel meta-analytic study therefore examined the effectiveness of school-based intervention programs in reducing adolescent psychological stress and which study, sample and intervention characteristics influence program effectiveness.

The school environment seems particularly suitable for intervention programs to reduce stress. Adolescents spend a substantial part of their time—on average six hours per school day—in school (Hofferth 2009), which makes the school an important context for cognitive development, as well as the development of social skills and emotional control, relevant for adequately dealing with stress (Resurrección et al. 2014). Since enhanced social and emotional functioning is beneficial for academic performance and school success (Zins et al. 2007), schools may benefit from implementing interventions that aim to improve social and emotional functioning. Moreover, school-based mental health services have been associated with a lower stigma and a greater utilization rate, especially among ethnic minority adolescents (Stephan et al. 2007). As such, school-based intervention programs provide a promising environment for low-threshold care, with the potential to also reach adolescents who are reluctant to search for care outside the school environment.

Over the past decade, various school-based intervention programs targeting adolescent stress have been developed. Some of these programs directly target stress, while other programs address stress as an indirect treatment aim. Moreover, to reduce stress and improve well-being of adolescents, these programs offer different approaches and apply various hypothesized mechanisms of change. For example, mindfulness (i.e., bringing non-judgmental attention to the present moment through meditation techniques and awareness exercises), relaxation exercises (e.g., progressive relaxation, muscle relaxation, visualization-based relaxation) and life skills training, comprising different cognitive-behavioral techniques (e.g., emotion regulation, problem-solving, conflict resolution), are often used (Rew et al. 2014). In terms of effectiveness, some studies on school-based stress intervention programs have yielded positive results (e.g., Jellesma and Cornelis 2012; De Wolfe and Saunders 1995; White 2012), whereas other studies indicated that interventions were not effective in reducing stress (e.g., Lang et al. 2017; Lau and Hue 2011; Terjestam et al. 2016).

The conflicting results of earlier studies are an important reason to conduct a meta-analysis to assess the effectiveness of school-based intervention programs in reducing adolescent psychological stress. Reportedly, there are only three reviews of the literature in this area (Feiss et al. 2019; Kraag et al. 2006; Rew et al. 2014). In their meta-analytic review, Kraag et al. (2006) investigated the effectiveness of school-based universal intervention programs targeting stress in children and adolescents. They demonstrated that these programs were effective in decreasing stress symptoms. Such promising results were supported by a narrative review on the effectiveness of stress reduction interventions in adolescents from community and clinical populations (Rew et al. 2014). In contrast, Feiss et al. (2019) showed that school-based stress prevention programs did not reduce stress symptoms in adolescents. In addition to generating conflicting findings, previous reviews bear a number of limitations. First, particularly the reviews by Kraag et al. (2006) and Rew et al. (2014) suffer from low quality of the included studies, limiting the robustness of their results. Second, Kraag et al. (2006) and Feiss et al. (2019) performed a traditional meta-analysis, a technique that does not allow the inclusion of multiple relevant effect sizes within studies. Third, Feiss et al. (2019) focused on school-based programs in the United States and based their meta-analysis on only four studies that assessed the effectiveness of such programs in terms of stress reduction. As such, their results do not inform us about the overall effectiveness of school-based intervention programs targeting stress in adolescents.

Because of these limitations, as well as the widespread implementation of school-based stress reduction programs and accompanying effectiveness studies since publication of the comprehensive review by Kraag et al. (2006), it is important to update the findings and conduct a new extensive meta-analysis. Consistent with Feiss et al. (2019), the present study included intervention programs in general adolescent populations (i.e., community samples) as well as selected adolescent populations (i.e., samples based on self-selection or screening, for instance on high stress or anxiety levels), given the potential of targeted interventions to be more efficient and to address problems early on (Offord 2000). Indeed, Feiss et al. (2019) demonstrated that targeted interventions yielded greater reductions in stress than universal interventions. Furthermore, the current study advances previous literature by performing a multilevel meta-analysis to fully exploit the available research data (i.e. allowing the inclusion of all relevant effect sizes per study) and generate more statistical power (Assink and Wibbelink 2016). This increased power ensures that extensive moderator analyses can be conducted. Investigating moderators is of crucial importance to better understand study results and to detect which interventions or components work best and which subgroups benefit most (Kraemer et al. 2002). This knowledge is necessary for the development of effective interventions and the selection of the best intervention for a specific population.

Based on previous meta-analyses on the effectiveness of intervention programs in youth, various study, sample and intervention characteristics may moderate program effectiveness. In terms of study characteristics, type of stress measured, publication year, publication status, study quality, the (in)dependence of authors, type of control condition, study design and timing of measurements were deemed important to consider. With regard to type of stress, effectiveness may vary across specific types of stress, such as school stress (e.g., pressure from study and worrying about grades or workload) or social stress (i.e., stress that stems from interpersonal relationships or from the social environment in general, such as the adolescents’ home life). Whether the study was performed recently or not might moderate the effectiveness, since the likelihood of reporting null-results has increased over the last two decades (Kaplan and Irvin 2015). However, previous research demonstrated no difference in publication year (Zoogman et al. 2015). Larger effects have been found for published versus unpublished studies (Conley et al. 2016), lower versus higher quality studies (Kraag et al. 2006), studies with quasi-experimental designs versus randomized controlled trials (RCTs) (Suter and Bruns 2009) and studies by researchers who developed the intervention program they studied versus independent researchers (Petrosino and Soydan 2005). Moreover, comparison with active versus passive control groups may impact findings of effectiveness studies (Feiss et al. 2019), and should therefore be considered. Furthermore, it is important to not only focus on post-intervention assessments, but also on follow-up assessments to investigate the long-term effects of school-based intervention programs.

Additionally, sample characteristics may affect the magnitude of the effects of school-based programs on stress reduction, for example age, gender distribution, socioeconomic status (SES) and ethnicity. A higher likelihood of effectiveness of intervention programs has been found in older versus younger samples and in samples with a higher proportion of females (Stice et al. 2009). Participants with low socioeconomic or minority backgrounds might respond differently to school-based intervention programs, possibly related to the implementation of the program. More specifically, schools in disadvantaged areas often suffer from various problems, such as high levels of unemployment, high staff turnover, poor facilities and lack of resources (Harris and Chapman 2004). This might make it more difficult to adequately implement intervention programs, resulting in lower effectiveness (Durlak et al. 2011). Furthermore, interventions in selected high-risk samples versus community samples (Stice et al. 2009), and targeted compared to universal intervention programs (Feiss et al. 2019) have been found to be more effective, suggesting that intervention programs generate more positive changes if problems are more severe at the start of the intervention. Moreover, it is possible that the selection method for including participants moderates program effectiveness. Inclusion based on self-selection might be more effective than inclusion based on screening, because it is likely that self-selected participants are more motivated to attend and actively participate in an intervention program (Stice et al. 2009).

Finally, there are some intervention characteristics that may affect the effectiveness of school-based intervention programs targeting psychological stress, including intensity, type of instructors and components and focus of the program. Previous meta-analyses demonstrated larger effects for less intensive interventions (Stice et al. 2009), and interventions delivered by external professionals (e.g. mental health professionals) as opposed to professionals working at the involved schools (Werner-Seidler et al. 2017). Moreover, techniques taught in intervention programs may affect effectiveness, with problem solving and emotional coping skills showing larger effects compared to relaxation techniques (Kraag et al. 2006). Whether or not the intervention directly addresses stress reduction might influence program effectiveness, because intervention programs with a direct focus on stress reduction may generate larger effects than interventions that address stress indirectly.

## Current Study

Given the increased number of school-based intervention programs targeting psychological stress in adolescents, and the limited knowledge on their overall effectiveness and factors influencing program effectiveness, it is important to conduct a new extensive meta-analysis. The current multilevel meta-analysis therefore aimed to determine the effectiveness of different school-based intervention programs in reducing psychological stress in adolescents. The second aim was to investigate which study (i.e., type of stress, publication year, publication status, study quality, study design, (in)dependence of authors, type of comparison condition, timing of measurements and time to follow-up), sample (i.e., age, gender, SES, ethnicity, target group and selection method), and intervention characteristics (i.e., intensity, type of instructor, components and focus of intervention) moderate the effectiveness of these programs.

School-based intervention programs were expected to reduce psychological stress in adolescents (Kraag et al. 2006). Larger effects were expected for published versus unpublished studies (Conley et al. 2016), for lower versus higher quality studies (Kraag et al. 2006), for studies by researchers who developed the intervention program they studied versus independent researchers (Petrosino and Soydan 2005), for studies with quasi-experimental designs versus RCTs (Suter and Bruns 2009) and for studies with active compared to passive control groups (Feiss et al. 2019). Larger effects were also expected for older, female samples (Stice et al. 2009), for samples with lower proportions of participants with low socioeconomic and minority backgrounds, and for selected samples, particularly based on self-selection (Stice et al. 2009). Furthermore, less intensive programs (Stice et al. 2009), intervention programs given by external professionals as opposed to professionals working at the involved schools (Werner-Seidler et al. 2017), having problem solving and emotional coping skills versus relaxation techniques as a component (Kraag et al. 2006), and that directly versus indirectly addressed stress reduction were expected to have larger effects. For type of stress, publication year and timing of measurements no hypotheses were formulated.

## Methods

The current study adhered to PRISMA guidelines (Moher et al. 2009).

### Selection Criteria

Available studies were searched that investigated the effects of school-based intervention programs on psychological stress in adolescents, meeting the following inclusion criteria: (1) studies had to evaluate the effectiveness of a school-based intervention program promoting psychosocial functioning (e.g., stress reduction, mental health, well-being, or coping skills), (2) studies had at least one psychological stress outcome, measured with self-report questionnaires, (3) studies had to target adolescents, with a mean age of 10 to 18 years old at the start of the intervention, (4) studies had to compare an experimental group and a control group, (5) studies had to include pre- and post-intervention assessment measures and/or follow-up assessment measures, (6) studies had to be written in English and (7) studies had to have available statistics suitable for performing meta-analyses (i.e., statistics to extract an effect size).

### Search Strategy

Through a systematic computer search, relevant publications were identified using the search engines Cumulative Index to Nursing and Allied Health Literature (CINAHL), PubMed, Education Resources Information Center (ERIC), PsycINFO and Cochrane. The search period was—since records began—up until June 2019, and four search terms were used. The search strings were “intervention* or program*” in combination with “stress or distress” in combination with “adolesc* or child or children or youth” in combination with “controlled clinical trial or controlled trial or random* or experiment* or comparison group* or controls or control condition* or control group* or control subject* or no treatment group* or waiting list or wait list or waitlist or treatment as usual or care as usual”. In addition, not statements were used to exclude studies that involved oxidative stress, distress syndrome, parenting stress, immunization, vaccination or venipuncture, studies that involved animals, infants, toddlers, preschool or kindergarten, studies about pregnancy, neonatal and prenatal, and study protocols, reviews and meta-analyses. Google Scholar was used to check the first 100 hits for missing relevant publications and to search for gray literature (i.e., unpublished work). Furthermore, a manual search through the reference lists of the identified publications, relevant review (Rew et al. 2014) and meta-analyses (Feiss et al. 2019; Kraag et al. 2006) was conducted.

### Coding of Studies

A detailed coding system was used to register study characteristics, outcome variables and moderators. All studies were coded by the first author. A subsample of the studies was double coded by either of two other researchers and responses of the two coders were compared (Inter-rater reliability (IRR) was 89.8% for a subset of 42.6% of the studies). Inconsistent responses were discussed with a fourth researcher to reach consensus. Effect sizes were coded for psychological stress (e.g., perceived stress, symptoms of stress). Positive effect sizes reflect improvements in functioning in the intervention group when compared to the control group. The following study, sample, and intervention characteristics were coded as moderators.

*Study characteristics* were type of stress outcome (school stress versus social stress), publication year (as a continuous variable), publication status (published or not published), study design ((cluster) RCT or quasi-experimental study, with RCT defined as randomly allocating participants to the experimental or control group, cluster RCT defined as randomly allocating groups of participants, and quasi-experimental design defined as a controlled study without random assignment of groups), type of comparison condition (passive control versus active control, with passive control defined as no intervention, regular school activities and waitlist control, and active control defined as treatment-as-usual and other interventions), the independence of the authors (whether or not the authors owned or (co-)developed the intervention), timing of measurements (whether the measurement was post-intervention or at follow-up, with post-intervention defined as measurement immediately after completion of the intervention and follow-up defined as measurement after the post-intervention measurement), time to follow-up (in weeks) since completion of the intervention (as a continuous variable), and study quality (as a continuous variable). The Quality Assessment Tool for Quantitative Studies (Thomas et al. 2004) was used to assess study quality, based on the characteristics selection bias, study design, confounders, blinding, data collection methods (validity and reliability) and withdrawals and dropout. Each variable was scored with 0 (not accounted for/missing), 1 (somewhat accounted for) and 2 (completely accounted for). Using these six variables, a total quality score was calculated for each study (range 0–12).

*Sample characteristics* were target group (non-selected or selected student samples, with non-selected students defined as samples of students from the general population and selected students defined as samples of students who self-selected or were selected based on prior screening), selection method (self-selection versus selection based on prior screening, such as participants self-selecting for an optional program or participants screened on high stress or anxiety levels), percentage of boys (as a continuous variable), percentage of low SES (i.e., low income, analyzed as a continuous variable), percentage of minorities (i.e., non-Caucasian, analyzed as a continuous variable) and mean age of the adolescents (if mean age was not reported, the midpoint of the age range was used, analyzed as a continuous variable).

*Intervention characteristics* were whether or not the intervention included the most often used stress reduction techniques (Rew et al. 2014), i.e., mindfulness (yes or no), relaxation exercises (yes or no) and cognitive-behavioral techniques (yes or no), intensity of the intervention (session duration multiplied by frequency of sessions; if session duration was reported as “a lesson”, the average of 45 min was used, analyzed as a continuous variable), type of instructors (specialized instructors or other instructors, including school personnel or researchers) and program target (whether stress reduction was a direct target of the intervention program or an indirect program target, based on the presence or absence of components that directly target stress management). Interventions with stress reduction as a direct program target included components to train mindfulness, yoga, relaxation or coping skills to manage stress, whereas interventions with stress reduction as an indirect program target included activities such as gardening or swimming, or components to train general coping or social skills. Program integrity (i.e., whether the intervention was applied according to protocol) was initially coded, but eventually not included as a moderator because few studies reported information about program integrity.

### Analysis of Effect Sizes

Using an online effect size calculator (Wilson n.d.), Cohen’s *d’*s were calculated for each effect size indicating the effectiveness of school-based intervention programs on psychological stress on the basis of differences between adolescents receiving an intervention program and adolescents in a control group. In most cases, Cohen’s *d* was calculated based on means and standard deviations (SD) or standard errors (SE). Group differences were computed for both pre- and post-intervention and pre-intervention *d*’s were subtracted from post-intervention *d*’s to account for baseline differences between groups (e.g., Van der Stouwe et al. 2014). When there were no means and SD/SE reported (13.8% of the total number of effect sizes), Cohen’s *d* was calculated based on mean difference scores, t-, F- or chi-square values. A small effect size was considered *d* = 0.20, a moderate effect size *d* = 0.50 and a large effect size *d* = 0.80 (Cohen 1988). Dummy variables were computed for the categorical moderators and continuous moderators were mean centered.

A three-level meta-analytic model was used in R to calculate an overall effect size and to conduct moderator analyses (Assink and Wibbelink 2016), thereby taking into account the dependency of multiple effect sizes from the same study (Van den Noortgate et al. 2013). Three levels of variance were included in the model: the sampling variance of each effect size (level 1), the within-study variance of effect sizes in the same study (level 2) and the between-study variance of effect sizes from different studies (level 3). The overall effect was estimated using an intercept-only model for psychological stress. The analysis was repeated after removal of outliers (i.e., extreme effect sizes, with an Interquartile Range (IQR) > 3) (Elbaum et al. 2000). Separate log-likelihood tests were performed to test if there was significant variance within (level 2) and between (level 3) studies (i.e., significant heterogeneity). If there was significant heterogeneity for at least one of the levels, moderator analyses were performed. In that case, possible moderators were included in the three-level intercept model (Assink and Wibbelink 2016). The Knapp and Hartung-method (Knapp and Hartung 2003) was applied, resulting in a decreased risk of Type 1-errors (Assink and Wibbelink 2016). Moderators were only included if there were at least three effect sizes for the specific moderator and at least one effect size per category of the moderator.

### Publication Bias

It is important to consider publication bias when conducting a meta-analytic study, because it is more likely that studies with positive results are published compared to studies that have negative or non-significant results, which could result in an underrepresentation of studies with minimal or negative effects. First, Rosenthal’s fail-safe test was performed, indicating no publication bias when the fail-safe *N* exceeds the critical value (derived by the formula 5 × *k* + 10, where *k* is the number of studies) (Rosenthal 1979). The critical value represents the number of studies with null results needed to make the overall result nonsignificant. Second, the funnel plot was visually inspected to detect asymmetry, which is an indication for publication bias. Third, in accordance with the Egger’s asymmetry test (Egger et al. 1997), a multilevel analysis was conducted with the sampling variance as a moderator to detect small study biases. Fourth, a trim and fill analysis was performed to test the influence of missing effect sizes on the results by repeating the meta-analysis after imputing the missing effect sizes (Duval and Tweedie 2000a, b).

## Results

### Study Selection

**Fig. 1:**
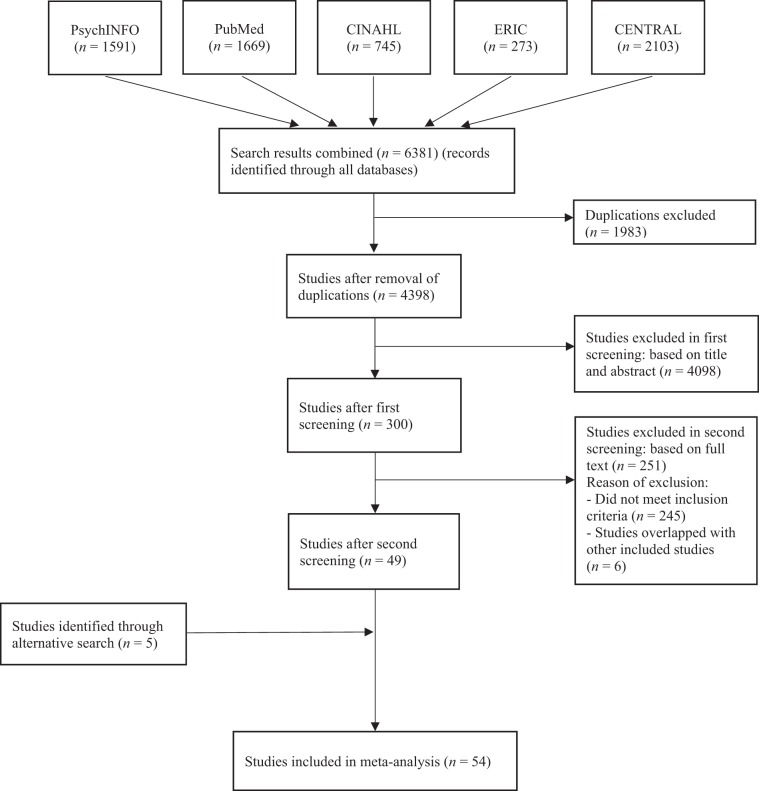
Flow chart

As displayed in the flowchart (Fig. 1), the electronic search identified 4398 unique hits for all databases after the removal of duplicates. After first selection by screening the title and abstract of the publications, 300 studies were potentially eligible. Following full text screening, 55 studies met the inclusion criteria. Of these 55 studies, six studies were excluded because they were earlier versions of included studies. The alternative search yielded 5 additional studies, which resulted in a final number of *k* = 54 included studies, reporting on analyses in 61 independent samples, yielding *N* = 123 effect sizes based on *N* = 9196 participants in an intervention group and *N* = 7279 participants in a control group.

All studies included in this meta-analytic review were issued between 1989 and 2019. Almost all studies were published, only 3 studies were not (i.e., dissertations). School or social stress, as an alternative to general stress, were examined in only 8 studies (based on 8 independent samples; 5 for school stress and 3 for social stress). Studies were (clustered) RCTs (37 studies, 41 independent samples) or quasi-experimental (17 studies, 20 samples). Most studies (32 studies, 36 samples) used a passive control group. About half of the studies was written by independent authors (26 studies, 27 samples). Only few studies (17 studies, 17 samples) included follow-up measurements, ranging from 4 to 48 weeks after the post-intervention assessment, with a mean of 17 weeks. Study quality ranged from 1 to 11, with a mean score of 6. Almost half (26 studies, 29 samples) included selected students, while the other half (28 studies, 32 samples) included non-selected, community sample students. Of the selected student samples, 16 were generated by screening and 13 by self-selection. Across samples, average age ranged between 10.3 and 17.7 years with an overall mean of 14.6 years. The mean percentage of boys was 41.4% (based on 58 samples), the mean percentage of minorities was 52.8% (based on 22 samples) and the mean percentage of low SES was 42.2% (based on 20 samples). With regard to the intervention programs, 47 studies (53 samples) directly focused on stress reduction, while the other studies did not. In terms of intervention components, mindfulness was included in 19 studies (21 samples), relaxation techniques in 21 studies (25 samples) and cognitive behavioral techniques in 25 studies (28 samples). The intensity of the intervention programs ranged from 100 to 9900 min, with a mean of 1015 min (based on 53 samples). Interventions were delivered by specialized instructors in 20 studies (22 samples) and by other instructors (e.g., school personnel, researchers) in 30 studies (35 samples). Details of the selected studies are provided in Table 1.

**Table 1:** Detailed description of the selected studies

Authors	*N*	Age range, mean age (SD), grade, gender, ethnicity^a^	Study design	Target group (selection)	Intervention	Program target (stress reduction)	Stress outcome^b^
Bennett and Dorjee (2016)	24	16–18 years, 17.70 (0.73) 11–12th grade, 58% boys	Quasi-experimental	Self-selected (voluntary)	Mindfulness-based stress reduction	Direct	Psychological stress (DASS, 7 items), perceived stress (body barometer, 1 item)
Bluth et al. (2016)	27	17.0 9–12th grade, 59% boys, 82% minorities	RCT	Self-selected (voluntary)	Learning to BREATHE	Direct	Perceived stress (PSS, 10 items)
Burckhardt et al. (2016)	63	15–18 years 10–11th grade, 61% boys	Cluster RCT	Screened (high depression, anxiety, stress levels)	Strong Minds	Direct	Stress levels (DASS, 7 items)
Butzer et al. (2017)	209	12.64 (0.33) 7th grade, 37% boys, 47% minorities	Cluster RCT	Community	Kripalu Yoga in the schools	Direct	Perceived stress (PSS, 10 items)
Campbell et al. (2019)	1007	13–19 years, 15.96 (1.17) 9–12th grade, 50% boys, 30% minorities	Quasi-experimental	Community	.b (the Mindfulness in Schools Project)	Direct	Perceived stress (PSS, 9 items)
Carreres-Ponsoda et al. (2017)	30	16–18 years, 16.80 50% boys	RCT	Self-selected (voluntary)	Mindfulness-based stress reduction	Direct	Perceived stress (PSS, 14 items)
Carter (2010)	64	13–16 years, 14.70 (0.74) 9–10th grade, 45% boys, 22% minorities	Cluster RCT	Self-selected (self-identified and identified by others)	The Best of Coping program	Direct	Stress appraisal of challenge, threat and resources (SAMA, 4, 7 and 3 items)
Cross et al. (2018)	2945	13.0 8–9th grade, 50% boys	Cluster RCT	Community	Friendly Schools Program	Indirect	Stress scores (DASS, 7 items)
Da Silva et al. (2019)	20	11–14 years, 12.10 (1.50) 70% boys	RCT	Screened (ADHD)	Swimming-learning program	Indirect	Perceived stress (PSS, 14 items)
De Anda (1998)	54	12–14 years, 13.00 30% boys, 53% minorities	Quasi-experimental	Self-selected (voluntary)	Cognitive-behavioral stress management program	Direct	Degree of experienced stress (ASCM, 4 items)
Dowling et al. (2019)	675	15–18 years, 15.87 (0.69) 5th grade, 50% boys	Cluster RCT	Community	MindOut program	Direct	Levels of symptoms related to stress (DASS, 7 items)
Ebrahmimi et al. (2015)	40	14–18 years, 16.48 (1.10) 100% boys	Quasi-experimental	Self-selected (voluntary)	Spiritual intelligence training	Direct	Stress scores (DASS, 7 items)
Eggert et al. (1995)	105	15.86 (1.01) 9–12th grade, 42% boys, 72% minorities	Quasi-experimental	Screened (suicide risk)	Personal growth class	Direct	Perceived stress and pressure from others (4 items)
Eslami et al. (2016)	126	16.33 (7.02) 0% boys	RCT	Community	Assertiveness training program	Direct	Stress levels (DASS, 7 items)
Fridrici and Lohaus (2009)	904	12–18 years 8–9th grade, 50% boys	Cluster RCT	Community	Stress prevention intervention	Direct	General stress (3 items)
Fung et al. (2019)	145	13–15 years, 13.99 (0.36) 9th grade, 32% boys, 97% minorities	RCT	Screened (depressive symptoms)	Learning to breathe	Direct	Perceived stress (PSS, 9 items)
Garcia et al. (2013)	41	13–16 years, 14.80 (0.72) 9–10th grade, 0% boys, 100% minorities	RCT	Self-selected (voluntary)	Project Wing’s Girl’s group	Direct	Perceived stress (PSS, 14 items), level of stress symptoms (DASS, 14 items)
Goodman and Newman (2014)	60	9th or 12th grade 0% boys	RCT	Self-selected (voluntary)	Digital storytelling	Direct	Experienced daily stress (ASQ, 31 items)
Hampel et al. (2008)	320	10–14 years, 11.70 (1.18) 5–8th grade, 50% boys	Quasi-experimental	Community	Anti-stress training	Direct	Interpersonal, academic stress (7 items)
Hiebert et al. (1989) Study 1	79	13–14 years 8th grade, 48% boys	Cluster RCT	Community	Progressive relaxation	Direct	Stress symptoms (SOSI, 59 items)
Hiebert et al. (1989) Study 2	22	17–18 years 11–12th grade, 63% boys	Quasi-experimental	Self-selected (elective module)	Progressive relaxation	Direct	Stress symptoms (SOSI, 59 items)
Jamali et al. 2016	100	13–14 years, 13.50 (1.01) 50% boys	RCT	Community	Life skills training	Indirect	Perceived stress (10 items)
Jellesma and Cornelis (2012)	54	8–13 years, 10.58 (1.58) 3th/6th grade, 65% boys	Cluster RCT	Community	Mind Magic Program	Direct	Psychological, mental stress (10-point scale)
Jose and Sajeena (2017)	60	8–10th grade	Cluster RCT	Self-selected	Yoga therapy	Direct	Perceived stress (PSS, 10 items)
Khalsa et al. (2012)	100	15–19 years, 16.80 (0.60) 11–12th grade, 58% boys, 10% minorities	Cluster RCT	Community	Yoga Ed program	Direct	Perception of stress (PSS, 10 items), social stress (BASC, 13 items)
Kiselica et al. (1994)	48	9th grade, 54% boys, 0% minorities	Cluster RCT	Screened (anxiety symptoms)	Stress inoculation training	Direct	Stress symptoms (SOSI, 118 items)
Kraag et al. (2009)	1437	10.30 (0.64) 5–6th grade, 50 % boys	Cluster RCT	Community	Learn Young, Learn Fair	Direct	Physiological, psychological stress symptoms (MUSIC)
Kuyken et al. (2013)	522	12–16 years, 14.80 (1.50) 70% boys, 28% minorities	Quasi-experimental	Community	Mindfulness in Schools Program	Direct	Perceived stress (PSS, 10 items)
Lai et al. (2016)	2304	14–16 years, 15.40 (1.00) 8–10th grade, 51% boys	Quasi-experimental	Community	The Little Prince is Depressed	Direct	Stress levels (DASS, 7 items)
Lang et al. (2017)	122	16.22 (1.12) 65% boys	Cluster RCT	Community	EPHECT coping training	Direct	Perceived stress (ASQ, 30 items)
Lau and Hue (2011)	48	14–16 years, 15.83 38% boys 0% minorities	Quasi-experimental	Self-selected (voluntary)	Mindfulness program	Direct	Perceived stress (PSS, 10)
Lee et al. (2018)	20	10–11 years, 10.50	RCT	Screened (emotional and behavioral problems)	Horticulture-related activities	Indirect	Stress levels: social, school stress (PSS, 10 items)
Livheim et al. (2015)	32	14–15 years 28% boys	RCT	Screened (depressive symptoms)	Acceptance and Commitment Therapy	Direct	Perceived stress (PSS, 10 items), stress levels (DASS, 7 items)
Manjushambika et al. (2017)	65	11–17 years, 14.50 42% boys	Quasi-experimental	Screened	Jacobson’s Progressive Muscle Relaxation	Direct	Educational stress (ESSA, 16 items)
Marsland et al. (2019)	70	8–14 years, 10.65 (1.49) 3–8th grade, 54% boys, 70% minorities	RCT	Screened (asthma)	I Can Cope	Direct	Perceived stress (PSS, 10 items)
Metz et al. (2013)	216	16.45 (0.95) 10–12th grade, 34% boys, 11% minorities	Quasi-experimental	Community	Learning to Breathe	Direct	Perceived stress level (1 item)
Noggle et al. (2012)	51	17.20 (0.70) 11–12th grade, 46% boys, 8% minorities	Cluster RCT	Community	Kripula yoga	Direct	Perceived stress (PSS, 10 items)
Norlander et al. (2005)	95	11.31 (1.09) 44% boys	Quasi-experimental	Community	Relaxation	Direct	Experienced stress levels (10 items)
Puolakanaho et al. (2019)	205	15.27 (0.39) 9th grade, 51% boys	RCT	Community	Youth COMPASS	Direct	Overall stress (1 item), school stress (4 items)
Quach et al. (2016)	149	12–15 years, 13.18 (0.72) 7–9th grade, 38% boys, 99% minorities	RCT	Community	Mindfulness meditation and hatha yoga	Direct	Perceived stress (PSS, 10 items)
Reiss (2013)	40	16–18 years, 17.25 (0.54) 12th grade, 65% boys	Quasi-experimental	Self-selected (voluntary)	Mindfulness meditation treatment	Direct	Perceived stress (PSS, 10 items)
Rentala et al. (2019)	60	16–19 years, 17.13, 0% boys	RCT	Screened (high stress levels)	Holistic group health promotion program	Direct	Stress levels (DASS, 7 items), educational stress (ESSA, 16 items)
Ruiz-Aranda et al. (2012)	147	13–16 years, 14.18 (0.64) 40% boys	RCT	Community	Emotional intelligence education program	Indirect	Social stress (BASC, 13 items)
Sibinga et al. (2013)	41	11–14 years, 12.50 7–8th grade, 100% boys, 95% minorities	RCT	Community	Mindfulness-based stress reduction	Direct	Perceived stress (PSS, 10 items)
Sibinga et al. (2016)	300	12.00 5–8th grade, 49% boys, 100% minorities	Cluster RCT	Community	Mindfulness-based stress reduction	Direct	Perceived stress (PSS, 6 items)
Silbert and Berry (1991)	145, 178	14–18 years, 15.00 10th grade, 50% boys, 70% minorities	Quasi-experimental	Screened, community	Suicide prevention unit	Indirect	Subjective experience of stress (SSS, 14 items)
Singhal et al. (2014)	19	13–18 years 9th grade, 0% boys	Quasi-experimental	Screened (sub-clinical depression)	Coping skills program	Direct	Academic stress (SAAS)
Singhal et al. (2018)	120	13–18 years 8th, 9th and 11th grade	Cluster RCT	Screened (sub-clinical depression)	Coping skills program	Direct	Academic stress (SAAS)
Solar (2013)	10	14–18 years, 16.00 (1.25) 9–12th grade, 70% boys, 30% minorities	RCT	Screened (emotional / learning disability or other health impairment)	Mindfulness meditation	Direct	Perceived stress (PSS, 10 items)
Terjestam (2011)	393	12–15 years, 13.90 7–9th grade, 48% boys	Cluster RCT	Community	Meditation based technique for stillness	Direct	General stress (3 items)
Terjestam et al. (2010)	119	13–14 years, 13.18 7th grade, 49% boys	Quasi-experimental	Community	Qigong	Direct	General stress (3 items)
Terjestam et al. (2016)	307	5–8th grade 52% boys	Cluster RCT	Community	Compas program	Direct	Stress levels (General Stress Scale, 3 items)
Van der Gucht et al. (2018)	390	13–20 years, 15.40 (1.20) 9–11th grade, 37% boys	Cluster RCT	Community	Mindfulness group training	Direct	Stress symptoms (DASS, 7 items)
Van Ryzin and Roseth (2018)	1449	7th grade 52% boys, 24% minorities	Cluster RCT	Community	Cooperation in the classroom	Indirect	Perceived stress (PSS, 4 items)
Zafar and Khalily (2015)	100	12–18 years 50% boys	RCT	Screened (high depression, anxiety, stress levels)	Didactic therapy	Direct	Stress levels (DASS, 14 items)

*DASS* depression anxiety stress scale, *RCT* randomized controlled trial, *PSS* perceived stress scale, *SAMA* stress appraisal measure for adolescents, *ASCM* the adolescent stress and coping measure, *ASQ* adolescent stress questionnaire, *SOSI* symptoms of stress inventory, *MUSIC* Maastricht University Stress Instrument for Children, *BASC* behavior assessment system for children and adolescents, *ESSA* education stress scale for adolescents, *SAA* scale for academic stress

^a^Percentage of minorities (i.e., non-Caucasian)

^b^Descriptions of authors

### Overall Effect

Test statistics for the overall effect can be found in Table 2. The overall effect size of school-based intervention programs on psychological stress was moderate (*d* = 0.543, *p* < 0.001), indicating that intervention programs are effective in reducing psychological stress. The heterogeneity test revealed that there was significant within-study and between-study variance (*p* < 0.0001).

**Table 2:** Result for the overall mean effect size

Outcome	*N s*tudies (samples)	*N* ES	*N* participants	Mean *d* (SE)	95% CI	*t*-value	LRT	% var	Fail-safe *N* (cv)
Psychological stress	54 (61)	123	16,475	0.543 (0.133)	0.279-0.806	4.082***	Level 2: 16.32***	Level 1: 1.5%	16,223 (280)
							Level 3: 100.41***	Level 2: 1.2%	
								Level 3: 97.3%	

*N studies (samples)* number of studies and independent samples, *N ES* number of effect sizes, mean *d* mean effect size Cohen’s *d*, *SE* standard error, *CI* confidence interval, *t-value* difference in mean *d* with zero, *LRT* likelihood-ratio test for level 2 and level 3, % *var* percentage of variance explained, *Fail-safeN* (cv) fail-safe number and Rosenthal’s critical value in parentheses

***p < 0.001

### Sensitivity Analysis

To account for the possible influence of outliers, the meta-analysis was repeated after removal of ten outliers (i.e., ten extreme positive effect sizes). This yielded a smaller but still significant effect on psychological stress (*d* = 0.276, *SE* = 0.064, *p<* 0.001).

### Publication Bias

**Fig. 2:**
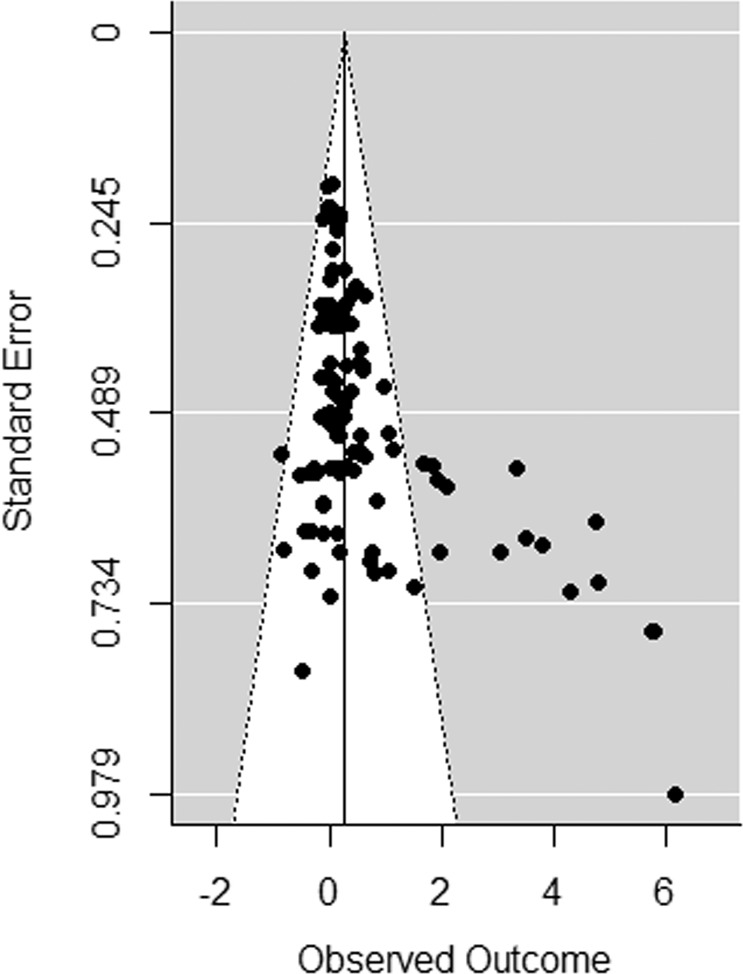
Funnel plot for psychological stress

The Rosenthal fail-safe test revealed that there was no indication of publication bias because the fail-safe *N* exceeded the critical value (see Table 2). However, the funnel plot (see Fig. 2) demonstrated asymmetry and the regression analysis of the sampling variance was significant (*p* < 0.001), indicating publication bias. The trim and fill analysis revealed that 26 effect sizes were missing on the left side of the distribution and results after imputation demonstrated a significant but very small overall effect on psychological stress (*d* = 0.068, *SE* = 0.011, *p* < 0.0001), thereby supporting the suggestion that studies with positive results are overrepresented, resulting in an inflation of the overall effect.

### Moderator Analyses

The results of the moderator analyses on psychological stress are reported in Table 3. Significant results are described here.

**Table 3:** Results for the moderator analyses on psychological stress

Moderator	*N* samples	*N* ES	*B*_0_ (95% CI)	*t*_0_	*B*_1_ (95% CI)	*t*_1_	*F*(df_1_, df_2_)	*p*
Study characteristics								
Type of stress							*F* (1, 13) = 4.754	0.048
School stress (RC)	5	10	2.739 (1.465–4.012)	4.646***				
Social stress	3	5	0.531 (−1.247 to 2.309)	0.646	−2.207 (−4.394 to −0.020)	−2.180*		
Publication year (continuous)	61	123	0.539 (0.276–0.801)	4.061***	0.020 (−0.013 to 0.054)	1.198	*F* (1, 121) = 1.436	0.233
Publication status							*F* (1, 121) = 1.557	0.215
Published (RC)	58	114	0.579 (0.310–0.848)	4.266***				
Not published	3	9	−0.210 (−1.433 to 1.012)	−0.340	−0.789 (−2.041 to 0.463)	−1.248		
Design							*F* (1, 121) = 0.566	0.453
(Cluster) RCT (RC)	41	74	0.613 (0.291–0.936)	3.765***				
Quasi-experimental	20	49	0.400 (−0.060 to 0.859)	1.723	−0.213 (−0.775 to 0.348)	−0.752		
Type of comparison condition							*F* (1, 121) = 1.336	0.250
Passive control (RC)	36	80	0.671 (0.328–1.014)	3.872***				
Active control	25	43	0.359 (−0.051 to 0.769)	1.732	−0.312 (−0.847 to 0.223)	−1.156		
Authors							*F* (1, 108) = 1.115	0.293
Independent (RC)	27	54	0.665 (0.292–1.038)	3.534***				
Dependent	25	56	0.380 (−0.003 to 0.763)	1.967	−0.285 (−0.820 to 0.250)	−1.056		
Time of measurements							*F* (1, 121) = 6.693	0.011
Post-intervention (RC)	44	93	0.522 (0.257–0.786)	3.906***				
Follow-up	17	30	0.672 (0.390–0.953)	4.724***	0.150 (0.035–0.265)	2.587*		
Time to follow-up (continuous)	17	30	0.964 (0.037–1.891)	2.131*	0.006 (−0.017 to 0.028)	0.504	*F* (1, 28) = 0.254	0.618
Study quality (continuous)	61	123	0.546 (0.280–0.812)	4.063***	0.019 (−0.096 to 0.134)	0.327	*F* (1, 121) = 0.107	0.744
Sample characteristics								
Target group							*F* (1, 121) = 7.065	0.009
Selected (RC)	29	58	0.908 (0.537–1.280)	4.844***				
Not-selected	32	65	0.234 (−0.104 to 0.572)	1.370	−0.674 (−1.177 to 0.172	−2.658**		
Selection method							*F* (1, 56) = 3.119	0.083
Screened (RC)	16	31	1.406 (0.676–2.135)	3.860***				
Self-selected	13	27	0.443 (−0.370 to 1.256)	1.091	−0.963 (−2.055 to 0.129)	−1.766		
% boys (continuous)	58	119	0.491 (0.245–0.737)	3.949***	0.000 (−0.009 to 0.010)	0.096	*F* (1, 117) = 0.009	0.924
% low SES (continuous)	20	41	0.233 (0.015–0.451)	2.163*	−0.000 (−0.004 to 0.004)	−0.034	*F* (1, 39) = 0.001	0.973
% minorities (continuous)	22	41	0.110 (0.007–0.213)	2.164*	0.001 (−0.001 to 0.003)	0.986	*F* (1, 39) = 0.972	0.330
Mean age (continuous)	61	123	0.548 (0.284–0.813)	4.101***	0.039 (−0.090 to 0.169)	0.601	*F* (1, 121) = 0.361	0.549
Intervention characteristics								
Component mindfulness							*F* (1, 121) = 1.644	0.202
Yes (RC)	21	31	0.360 (−0.020 to 0.740)	1.874				
No	40	92	0.633 (0.341–0.926)	4.281***	0.274 (−0.149 to 0.696)	1.282		
Component relaxation							*F* (1, 121) = 3.288	0.072
Yes (RC)	25	42	0.828 (0.422–1.233)	4.040***				
No	36	81	0.345 (0.008–0.682)	2.025*	−0.483 (−1.010 to 0.044)	−1.813		
Component cognitive-behavioral							*F* (1, 121) = 2.613	0.109
Yes (RC)	28	78	0.772 (0.388–1.156)	3.983***				
No	33	45	0.345 (−0.010 to 0.700)	1.923	−0.427 (−0.950 to 0.096)	−1.617		
Intensity (continuous)	53	100	0.489 (0.222–0.756)	3.632***	−0.000 (−0.000 to 0.000)	−0.256	*F* (1, 98) = 0.065	0.799
Type of instructors							*F* (1, 110) = 2.445	0.121
Specialized (RC)	22	47	0.603 (0.271–0.934)	3.601***				
Other	35	65	0.270 (0.010–0.53)	2.056*	−0.333 (−0.754 to 0.089)	−1.564		
Program target							*F* (1, 121) = 0.089	0.765
Direct (RC)	53	111	0.559 (0.274–0.844)	3.879***				
Indirect	8	12	0.440 (−0.289 to 1.170)	1.196	−0.118 (−0.902 to 0.665)	−0.299		

*N samples* number of independent samples, *N ES* number of effect sizes, *B*_0_ mean effect size Cohen’s *d,CI* confidence interval, *B*_1_ estimated regression coefficient, *t-values* difference in mean *d* with zero, *F-value* omnibus test of regression coefficients, *pp*-value of omnibus test, *RC* reference category

*p < 0.05; **p < 0.01; ***p < 0.001

#### Study characteristics

The type of stress and timing of measurements moderated the effect, yielding significant effects for school stress, but not for social stress. Larger effects were found at follow-up compared to post-intervention.

#### Sample characteristics

The target group moderated the effect, demonstrating significant effects in selected student samples, but not in non-selected samples.

#### Intervention characteristics

No intervention characteristics moderated the effects.

## Discussion

In order to prevent adverse adolescent development resulting from high levels of stress, it is important that heightened stress levels are addressed early on. Although various school-based intervention programs have been implemented to reduce adolescent stress, little is known on their overall effectiveness and factors influencing their effectiveness as previous reviews investigating stress reduction in adolescents through school-based intervention programs have generated conflicting and selective findings (Feiss et al. 2019; Kraag et al. 2006; Rew et al. 2014). In this comprehensive multilevel meta-analytic review, the extent to which school-based intervention programs are effective in reducing adolescent psychological stress was examined. In addition, study (e.g., publication status, study design), sample (e.g., age, target group) and intervention characteristics (e.g., intensity, components) were investigated as moderators of effectiveness. The current meta-analysis showed that school-based intervention programs had a moderate overall effect on reducing psychological stress. Significant program effects were only observed in selected student samples and not in community samples. Based on a subsample of studies with specific measures of school and social stress (instead of or in addition to general measures of stress), interventions were particularly effective in reducing school stress, not social stress. In addition, larger effects were found at follow-up compared to post-intervention.

The overall finding that school-based intervention programs are effective in reducing adolescent stress is consistent with the conclusion of Kraag et al. (2006). However, Kraag et al. (2006) only focused on universal interventions delivered to students from the general population, showing that this group benefits from school-based interventions. In contrast, based on the moderator analyses, the present study suggests that school-based interventions targeting psychological stress are not effective in community samples, and that only selected students benefit from such interventions. These contrasting findings may be explained by the difference between the two studies in age groups. In Kraag et al. (2006) participants were between 9 and 14 years, while in the current study participants were between 10 and 18 years old (with a mean age of 15 years). A difference in effectiveness of universal programs between age groups might be explained by the changing importance of the class environment with age. Specifically, while primary school students spend every day with the same teacher and classmates with whom they generally develop close relationships and feel comfortable (Coffey 2013), students in secondary school typically develop fewer close relationships, especially with their teachers (Tobbell and O’Donnell 2013). Consequently, the class environment in secondary school may be less safe to learn new skills than in primary school (i.e., for older compared to younger adolescents). This may result in smaller effectiveness of universal programs in secondary schools or older adolescents. Indeed, for a universal school-based intervention program targeting anxiety, it has been demonstrated that effectiveness was lower for secondary compared to primary school students (Barrett et al. 2005). Additionally, the contrasting findings may be explained by methodological differences between Kraag et al. (2006) and the current study. First, Kraag et al. (2006) included both psychological and physiological stress symptoms. Possibly, large positive effects in terms of physiological outcomes, which are not included in the present study, may explain the difference in findings. Second, Kraag and colleagues computed the effect sizes as the difference in mean change from pretest to posttest between the treatment and control group. This is in contrast with the current study that used group differences (i.e., between the intervention and control group) for both pre- and post-intervention assessments, thus correcting for pre-intervention differences between groups. The results of Kraag and colleagues could be influenced by baseline differences between the intervention and control groups. Third, Kraag et al. (2006) showed publication bias for the effect on stress symptoms, which suggests that their effect on stress reduction was overestimated.

Finding only significant effects for selected student samples compared to non-selected samples is in line with earlier research on school-based stress reduction programs (Feiss et al. 2019). Moreover, previous studies demonstrated that targeted programs were more effective than universal school-based depression programs (Werner-Seidler et al. 2017). This is probably associated with the difference in baseline symptoms between students of the general population and selected students, with selected students demonstrating higher levels of problem severity. Recent research demonstrated that program improvement is more evident in students with a high level of baseline problems (Stjerneklar et al. 2019). Moreover, selected students may be more motivated to actively participate in the intervention than students from the general population because they experience distress about their problems, resulting in larger program effects (Stice et al. 2009).

Based on a subsample of included studies, the results indicated that school-based intervention programs particularly affected school stress (e.g., study pressure, workload, worry on grades) and not social stress. A possible explanation is that adolescents can relate more to study-related stress and may apply their school-learned skills particularly in the context of study situations rather than social situations. Additionally, in the present study, three of five studies (i.e., 7 of 10 effect sizes) that measured school stress examined an intervention program containing a specific component on dealing with academic stress, while none of the three studies that measured social stress examined an intervention program with a specific component on dealing with social stress. The matching of a specific program component with a similar outcome variable might explain the observed larger effects for school stress. Yet, as perceived school-related stress affects many adolescents worldwide (Klinger et al. 2015), it is promising that school-based interventions have the potential to alleviate school-related stress. At the same time, given the limited number of studies and accompanying effect sizes with specific measures of school stress and social stress (i.e., 15 effect sizes), this finding should be interpreted with caution. To better understand the impact of intervention programs on stress reduction, future studies are recommended to include measures of stress that match the type of stress targeted in the intervention program studied.

Follow-up assessments yielded larger effects in terms of reductions in psychological stress than assessments at post-intervention. On the one hand, this might indicate a sleeper effect, i.e., improved longer term outcomes, which has been suggested for reductions in depressive symptoms that are only expected at a later stage when adolescents have experienced challenging situations (Spence and Shortt 2007). This sleeper effect may also apply to reductions in psychological stress following universal and selective interventions, as larger effects at follow-up were likely in both selected and non-selected student samples. Yet, recent meta-analyses demonstrated marginal evidence for a sleeper effect of psychotherapy interventions (Flückiger and Del Re 2017). On the other hand, finding larger effects at follow-up may be due to differences in sample composition between studies with and without follow-up assessments. In the current study, studies with follow-up assessments included more females and more selected student samples than studies with only post-intervention measurements (68% vs. 56% females and 63% vs. 42% selected samples), characteristics that have been associated with larger program effects (Stice et al. 2009).

### Limitations

Several limitations need to be considered in this meta-analysis. First, although efforts were made to minimize publication bias by including gray literature and contacting authors of included studies for unpublished work, publication bias was indicated and might have inflated the overall estimates. Even though the validity of the available methods to detect publication bias is questioned for multilevel meta-analyses (Assink and Wibbelink 2016), making these specific results difficult to interpret, it is important to keep in mind that the program effects might be overestimated.

Second, extreme positive effect sizes (i.e., outliers) were observed. A sensitivity analysis was therefore conducted to account for their possible influence, by repeating the meta-analysis after the removal of outliers. Although the effect on psychological stress was smaller after correction, it remained significant. This indicates that the outliers moderately overestimated the overall effect. To further understand the impact of outliers, the included studies with extreme scores were examined. These studies were all based on selected rather than community student samples, had higher proportions of female participants and more often included follow-up assessments; factors that were found to be associated with larger effects in the present study and in previous research (Stice et al. 2009). As such, extreme scores seem to result from combinations of characteristics associated with larger effects.

Lastly, limited information was available for some of the study, sample, and intervention characteristics, including percentage of SES, percentage of minorities and program integrity. This limited the possibilities to conduct moderator analyses. It is important that future intervention studies report sufficient information about the study, sample, and intervention characteristics in order to be able to determine what works for whom in school-based intervention programs targeting stress.

### Recommendations for Future Research

The present multilevel meta-analytic study evaluated the effectiveness of school-based intervention programs targeting adolescent psychological stress. In addition to new insights into the effectiveness of school-based intervention programs targeting psychological stress in adolescents, the current study generates recommendations for future research. It is recommended that future studies report information about program integrity (i.e., whether the intervention program was implemented as originally planned). As non-significant or negative results may be caused by incorrect program implementation, and not by an ineffective program, information about the program implementation is necessary to draw correct conclusions about the effectiveness of intervention programs. Furthermore, although no significant effects were found for universal interventions, contradicting earlier findings (Kraag et al. 2006), it is still important to examine them. Universal interventions reach larger groups of adolescents, including adolescents with (emerging) problems who do not search for care outside the school environment. It is of great importance to identify if adolescents with (emerging) problems benefit from universal interventions, or to examine how universal intervention programs can be adjusted to achieve the desired results for adolescents in need (e.g., improvement in functioning, effective referral). Overall, further research is necessary to identify the working mechanisms of effective school-based intervention programs targeting adolescent stress, for specific types of stress (e.g., school stress, social stress).

## Conclusion

Previous reviews have investigated the reduction of adolescent stress through school-based intervention programs, however, these studies have yielded conflicting and selective findings. To overcome these limitations, the present multilevel meta-analytic study examined the effectiveness of school-based intervention programs in reducing psychological stress in adolescents and examined study, sample and intervention characteristics as moderators of effectiveness. The current study showed that school-based intervention programs were effective at reducing adolescent psychological stress, particularly for selected student samples. Furthermore, findings based on a small subsample of studies suggest that interventions were particularly effective in reducing school stress rather than social stress. Lastly, larger effects were found at follow-up compared to post-intervention, although this finding likely results from the sample composition of studies including follow-up assessments. Since heightened stress is an increasing mental health issue among adolescents (Walburg 2014), it is important that governments and schools are aware of the availability and potential of school-based intervention programs to reduce psychological stress in adolescents, and implement such programs in practice. This pertains particularly to interventions directed at students who self-select or enroll following a screening, as they benefit most from such interventions. School-based intervention programs aimed at reducing adolescent stress are scarce compared to programs aimed at reducing anxiety or depressive symptoms (Feiss et al. 2019). Yet, teaching adolescents skills to adequately deal with stress is of interest to both adolescents and schools, since addressing psychological stress through school-based intervention programs may prevent emerging mental health issues that likely also affect school performance.
